# Approach to the Triple Negative Breast Cancer in New Drugs Area

**Published:** 2016-04-01

**Authors:** Mehrzad Mirzania

**Affiliations:** Hematology and Medical Oncology Department, Cancer Research Center, Cancer Institute of Iran, Imam Khomeini Hospital Complex, Tehran University of Medical Sciences, Tehran, Iran

**Keywords:** Triple negative breast cancers, PARP inhibitors, GPNMB, Src family kinase, PI3K/AKT/mTOR pathway

## Abstract

Triple negative breast cancers (TNBCs) are associated with aggressive course, higher rates of visceral and central nervous system metastases and lower survival rate than hormone receptor positive. Once metastasis has occurred, a median survival was approximately one year. Currently, chemotherapy in TNBC is similar to other HER2- negative breast cancers but in the near future, it will revolutionize. TNBCs are quite heterogeneous based on biomarkers and genetic variations. The series of new drugs have been tried; in this article, platinum, anti-epigenetic drugs, PARP inhibitors, epidermal growth factor receptor inhibitor, Src family kinase inhibitor, anti androgen, glycoprotein Non-metastatic melanoma B (gpNMB) antibody, LHRH conjugated to cytotoxic drugs and inhibition of the PI3K/AKT/mTOR pathway will be explained. What is the optimal therapy for TNBC patients? It is still not clear but it seems that the road map according to biological and genetic markers is taking shape.

## Introduction

 In definition, triple negative breast cancer to mean cancers that have ≤1 percent expression of ER and PR as determined by immunohistochemistry (IHC), and that are HER2 either 0-1+ by IHC, or 2+ with fluorescence in situ hybridization (FISH) negative.^1^

Triple negative breast cancer accounts for approximately 20 percent of breast cancers diagnosed worldwide. Up to 20 percent of patients with triple negative breast cancer harbor a breast cancer gene (BRCA) mutation, particularly in BRCA1. Premenopausal status as well as three or more births and obesity were associated with an increased risk of triple negative breast cancer.^2^^,^^3^ Studies suggest that triple negative breast cancers present aggressively with rapid growth and are more likely to be diagnosed clinically rather than mammographically. Separate subtypes of triple negative breast cancer have been characterized by gene expression including two basal-like subtypes (BL1 and BL2), an immunomodulatory, mesenchymal, mesenchymal stem-like, luminal androgen receptor (LAR) subtypes and two additional subtypes that include claudin-low and interferon-rich.^4^^,^^5^^,^^6^ Basal breast cancer is characterized by a unique cluster of genes that includes the epidermal growth factor receptor (EGFR, also called HER1), basal cytokeratins 5/6 and c-Kit.^7^ About 70% of TNBC are basal type.

TNBC is usually high-grade and is associated with poorer prognosis in comparison with other subtypes of breast cancer but the principles for the surgical, radiation therapy and neoadjuvant or adjuvant chemotherapy options are similar to the other breast cancer phenotypes. Many studies have been conducted and their results can help us to overcome this problem.

**Role of platinum agents**

Pathologic complete response (pCR) with neoadjuvant chemotherapy is higher in TNBC (22 versus 11 percent, p = 0.034). pCR associated with improvement in disease-free survival (DFS);^8^ despite of this, TNBC had poorer overall outcome compared with patients with luminal subtypes or HER2- positive breast cancer because additional adjuvant treatments are not available for them.^9^^,^^10^

The administration of neoadjuvant carboplatin and standard anthracycline plus taxane-based chemotherapy for all patients with triple-negative breast cancer is not advised. In this case, there are three studies: In the German GeparSixto trial and in CALGB 40603 (Alliance), the addition of carboplatin resulted a significantly higher pCR rate and also a significantly higher rate of treatment related toxicities.^11^^,^^12^ In the third study (ISPY-2), weekly T plus dose-dense AC with or without regimen consisting of carboplatin (every three weeks for four cycles) and veliparib. None of these studies were sufficiently powered to detect the specific addition of carboplatin that significantly improves RFS or OS.

In one clinical trial, 67 women with BRCA1 mutation were treated with cisplatin for four cycles as neoadjuvant treatment. The pathologic complete response was achieved in 67 % of patients.^13^ In the TNT trial, docetaxel was directly compared with carboplatin in patients with metastatic or local recurrent TNBC.

In this multicenter phase III trial, 376 patients were randomized to either carboplatin (AUC 6) q3 weekly for 6 cycles or docetaxel 100 mg/m2 q 3 weekly for 6 cycles. Upon progression, patients were encouraged to cross-over to the other arm. The primary end-point was overall response rate (ORR) at cycle 3 or 6, ORR were similar, except in women with a known BRCA1/2 mutation, in whom there was a significantly higher response rate with carboplatin, the two treatment arms were 8% BRCA 1/2 mutation. It is unclear whether survival outcomes will be improved by the routine addition of platinum agents over other standard treatments.

**EGFR**

The epidermal growth factor receptor (EGFR/HER1) is overexpressed in some TNBC tumors. In the TBCRC 001 trial (a randomized phase II trial), patients with metastatic TNBC received anti-EGFR antibody cetuximab (400 mg/m2 load then 250 mg/m2 per week) alone with carboplatin (area under the curve of 2, once per week IV) added after progression or as concomitant therapy from the beginning. Response rate (RR) was the primary end point. In 102 patients with TNBC, RRs were 6% (two of 31) to cetuximab and 16% (four of 25) to cetuximab plus carboplatin after progression. RR was 17% for those treated with cetuximab plus carboplatin from the beginning (12 of 71). 31% of patients responded or had prolonged disease stabilization. The cetuximab plus carboplatin regimen was well tolerated, but both TTP and OS were short at 2.1 months (95% CI, 1.8 to 5.5 months) and 10.4 months (95% CI, 7.7 to 13.1 months), respectively.^14^

**Epigenetic**

The term epigenetic describes dynamic alterations in a cell that are caused by external or environmental factors that switch genes on and off and affect how cells read genes without changes in the DNA sequence. In other words, non-genetic factors cause the cell's genes to behave differently. Examples of mechanisms that produce such changes are DNA methylation and histone modification, each of which alters how genes are expressed without altering the underlying DNA sequence. Addition of histone deacetylase (HDAC) inhibitor vorinostat to endocrine therapy in the setting of endocrine-resistant disease appears to restore sensitivity in selected patients, a hypothesis that is being carried forward in the setting of ER-negative breast cancer.

Currently, these agents such as DNA methyltransferase and histone deacetylase inhibitors should not be used for the treatment of TNBC outside of a clinical trial.

**PARP (poly adenosine diphosphate-ribose polymerase) inhibitors**

PARP is leading to cell recovery from DNA damage. BRCA function can also repair double-strand DNA breakage. PARP inhibitors may be particularly useful in BRCA-mutated breast cancer. This is a hypothesis that inhibition of PARP in combination with DNA-damaging chemotherapy may be overcome to BRCA function.

In one study with olaparib, women with BRCA1 or BRCA2 mutations and recurrent advanced breast cancer were assigned to two sequential cohorts in a phase 2 study. The first cohort (n=27) was given continuous oral olaparib at the maximum tolerated dose (400 mg twice daily), and the second (n=27) was given a lower dose (100 mg twice daily). The primary efficacy endpoint was objective response rate (ORR). Patients had been given a median of three previous chemotherapy regimens; ORR was 11 (41%) of 27 patients in 400 mg twice daily and six (22%) of 27 in 100 mg twice daily. Toxicities were mainly at low grades. The results of this study provide positive proof of concept for PARP inhibition in BRCA-deficient breast cancers and show a favorable therapeutic index for a novel targeted treatment strategy in patients with tumors that have genetic loss of function of BRCA1-associated or BRCA2-associated DNA repair.^15^

Veliparib was tested in combination with temozolomide in 41 women with advanced triple negative breast cancer in a single arm phase II study. In the subgroup with BRCA mutations, the overall response and clinical benefit rates were 37.5% and 62.5 %, respectively.

Iniparib was evaluated in a phase III trial in which 519 women with pretreated metastatic triple negative breast cancer were randomly assigned to gemcitabine plus carboplatin with or without iniparib (5.6 mg/kg, intravenously, days 1, 4, 8 and 11) every 21 days. The median PFS was modestly but not statistically significantly longer in the iniparib group (median 5.1 versus 4.1 months), and overall survival was similar (11.8 versus 11.1 months).

**Src family kinase**

Src family kinase is a family of non-receptor tyrosine kinases that includes nine members. Gene expression profiling has suggested that triple negative breast cancer might be sensitive to inhibition of the proto-oncogene Src. Dasatinib is a potent inhibitor of Src-family kinase, that may have clinical benefit in this setting.^16^ Cell lines belonging to the mesenchymal-like subtypes (M and MSL) were more sensitive to dasatinib.^4^

**Antiandrogen**

Enzalutamide has shown activity in the subset of women with advanced triple negative breast cancer whose tumors express the androgen receptor (AR). The results of the phase II single-arm trial (abstract 1003) were presented by Tiffany Traina at the 2015 American Society of Clinical Oncology (ASCO). The Annual Meeting was held on May 29 to June 2 in Chicago. The trial enrolled 118 women with AR-positive triple negative breast cancer. More than 50% of the patients received enzalutamide as either a first- or second-line therapy for their metastatic disease. Patients were treated with 160 mg of enzalutamide daily until disease progression. The trial met its primary endpoint of clinical benefit at 16 weeks of therapy. Of the 75 patients who could be evaluated, 35% achieved a clinical benefit. There were two complete responses and seven partial responses. The clinical benefit rate at ≥ 24 weeks was 29%. The median PFS was 14.7 weeks. About 20% to 40% of triple-negative breast tumors expressed AR.

**Glembatumumab vedotin (GV)**

Glycoprotein Non-metastatic Melanoma B (gpNMB) is a negative prognostic marker that overexpressed in 40% of TNBC, gpNMB can promote angiogenesis, migration, invasion, and metastasis.^17^ Glembatumumab vedotin is a gpNMB-specific monoclonal antibody conjugated to the potent cytotoxic monomethyl auristatin E. By definition, overexpression of gpNMB is positivity of more than 5% of cell in IHC. In one phase II study, ORR was significantly better (40 versus 0 %) in subgroup of patients that overexpressed gpNMB more than 25 % in IHC.^18^

**LHRH conjugated to cytotoxic**

Specific receptors for LHRH (luteinizing hormone-releasing Hormone) are detected in the pituitary gland and in tissue of male and female reproductive organs. They are not expressed in other tissues or in benign tumors, but these receptors have been detected in breast, prostatic, ovarian and endometrial cancer cells. Up to 49% of human surgical specimens of TNBC were LHRH receptor expression by immunohistochemistry. AEZS-108 is a hybrid molecule that consists of doxorubicin linked to the LHRH agonist. In one preclinical study, AEZS-108 has a toxic effect on the LHRH-R positive TNBC cell line.^19^ This in vitro study shows that LHRH-receptors can be used as homing sites for cytotoxic agents.

**Inhibition of the PI3K/AKT/mTOR Pathway**

The PI3K/Akt/mTOR pathway is a survival pathway that is activated in many types of cancer. Mechanisms for pathway activation include loss of tumor suppressor PTEN function, amplification or mutation of PI3K, amplification or mutation of Akt, activation of growth factor receptors and exposure to carcinogens. When this pathway is activated, signaling through Akt extent to some other pathways including PI3K/Akt/mTOR pathway can provide a cancer cell survival, metabolism, proliferation, motility, migration, invasion, and angiogenesis.^20^ This signaling pathway is seen in approximately 10 % of TNBC patients, especially in mesenchymal and LAR molecular subtypes.^21^ Ipatasertib is serine/threonine kinase AKT inhibitor. LOTUS is an ongoing randomized phase II study to evaluate the efficacy of ipatasertib combined with paclitaxel, the primary endpoint of this article is PFS.

## CONCLUSION

 At present, we have similar chemotherapeutic options for TNBC as other HER2- negative breast cancer tumors, of course with poorer outcome. Some new drugs are emerging such as LHRH conjugated to cytotoxic in patients who are LHRH receptor positive (49% of TNBC), gpNMB- antibody conjugated to cytotoxic drugs in patients who overexpressed gpNMB (40% of TNBC), anti androgen in patients who are AR positive (20%-40% of TNBC), Src family kinase inhibitors in patients who are mesenchymal-like subtypes, EGFR antibody in patients who are basal-like subtype (50%-71% of TNBC), PARP inhibitors and platinum agents in patients who are BRCA mutations (20% of TNBC), HDAC inhibitors and inhibition of the PI3K/AKT/mTOR pathway (10% of TNBC). In the future, it seems that we can plan the treatment of TNBC for each patient based on genetic and biological markers. To achieve this goal, we must await the results of future studies.
